# Associations between Meteorological Factors and Aseptic Meningitis in Six Metropolitan Provinces of the Republic of Korea

**DOI:** 10.3390/ijerph13121193

**Published:** 2016-11-30

**Authors:** Yadav Prasad Joshi, Eun-Hye Kim, Jong-Hun Kim, Ho Kim, Hae-Kwan Cheong

**Affiliations:** 1 Department of Social and Preventive Medicine, Sungkyunkwan University School of Medicine, 2066 Seobu-ro, Jangan-gu, Suwon, Gyeonggi-do 16419, Korea; yadavjoshi@gmail.com (Y.P.J.); dodendlqtnf@gmail.com (E.-H.K.); kimjh32@skku.edu (J.-H.K.); 2 Department of Biostatistics and Epidemiology, Graduate School of Public Health, and Institute of Public Health and Environment, Seoul National University, 1 Gwanak-ro, Gwanak-gu, Seoul 08826, Korea; hokim@snu.ac.kr

**Keywords:** climate, *Enterovirus*, seasonality, sea surface temperature, quasi-Poisson models

## Abstract

We assessed the association between climate factors and a number of aseptic meningitis cases in six metropolitan provinces of the Republic of Korea using a weekly number of cases from January 2002 to December 2012. Generalized linear quasi-Poisson models were applied to estimate the effects of climate factors on the weekly number of aseptic meningitis cases. We used generalized additive and generalized additive mixed models to assess dose–response relationships. A 1 °C increase in mean temperature was associated with an 11.4% (95% confidence interval (CI): 9.6%–13.3%) increase in aseptic meningitis with a 0-week lag; a 10 mm rise in rainfall was associated with an 8.0% (95% CI: 7.2%–8.8%) increase in aseptic meningitis with a 7-week lag; and a 1 mJ/m^2^ increase of solar radiation was associated with a 5.8% (95% CI: 3.0%–8.7%) increase in aseptic meningitis with a 10-week lag. Nino3 showed positive effects in all lags, and its one unit increase was associated with an 18.9% (95% CI: 15.3%–22.6%) increase of aseptic meningitis at lag 9. The variability in the relationship between climate factors and aseptic meningitis could be used to initiate preventive measures for climate determinants of aseptic meningitis.

## 1. Introduction

Enteroviruses (EVs) are non-enveloped RNA viruses of the family *Picornaviridae*. Human EVs are sub-grouped into poliovirus, coxsackievirus group A (CAV), coxsackievirus group B (CBV), echovirus, and the newer EV serotypes 68 to 71 [1,2]. They spread mainly by the fecal–oral and oral–oral routes or through direct contact, water, food, or soil contaminated with feces [1,3]. Enteroviral diseases are found year-round in tropical and semi-tropical climates and primarily in the summer and fall months in temperate regions [1,2,4]. Prevalence and transmissibility vary by viral strain, climate, season, geography, crowding, and socio-economic status [4,5,6,7]. Different EVs can cause similar symptoms while a single EV can cause a range of symptoms, from undifferentiated febrile illness to undisguised aseptic meningitis (AM) [2,8]. AM is primarily characterized by inflammation of the meninges of the central nervous system. The major causative agents belong to the CAV (serotypes 2, 4, 10, 24), CBV (serotype 5), and echovirus (serotypes 4, 6, 9, 12, 13, 25, 30, and 33) [5,7,8,9]. AM outbreaks predominantly occur among children during the summer months in the Republic of Korea (Korea), Taiwan, and China [5,8,9]. The seasonality of AM suggests that meteorological factors might influence the spread and distribution of EVs because their survival and stability are generally influenced by environmental and climatic variables [10,11]. 

Climatic variability and inter-annual climate change have influenced the incidence of several microbial water- and food-borne diseases [12]. The variations of local weather conditions also influence the occurrence and hospital admissions of non-infectious such as respiratory and cardiovascular diseases [13,14]. The EI Niño–Southern Oscillation (ENSO) and Indian Ocean Dipole (IOD) are well studied climate phenomena associated with the largest periodic climate variability in different parts of the world [12,15,16], including Korea [17,18]. Researchers have observed their effects on the incidence of enteroviral and other infectious diseases [15,19,20,21,22,23]. 

In Korea, enteroviral AM has become a serious public health problem among children [24,25]. Outbreaks of AM occurred approximately every three years since 1990 [5,24,26]. In 1996–2001, the average annual incidence of AM among children under 15 was 3.5 per 1000 [25]. In 1999–2003, 603 isolates of echovirus (13) and CAV (serotypes 24 and 9) were identified in 2939 patients with AM and other enteroviral diseases [7]. In the 2008 outbreak, 67.7% of samples from 758 AM patients were positive for *Enterovirus*, and 98% of them were from children younger than 15 years [5]. A variety of EVs have been identified as the predominant causative agents in each summer epidemic of Korea [5,7]. For example, echovirus (9) in 1993, echovirus (serotypes 30 and 6) in 1998, echovirus (13) and CAV (24) in 2002, echovirus (18) and CBV (5) in 2005, and echovirus (serotypes 6 and 30) in 2008 [5,7,26,27]. Previous Korean studies have mostly focused on the descriptive, seroepidemiology, and molecular biology of EVs [25,26,27]. However, there is a need for in-depth statistical analysis of the relationship between meteorological variables and AM cases in Korea. It is not known how different local weather factors and oceanic climate variables are associated with the ecological distribution of AM. In this study, we aimed to establish the relationships between meteorological variables and the weekly sum of AM cases in six metropolitan provinces of Korea.

## 2. Materials and Methods

### 2.1. Study Areas

Korea is located in a transitional zone on the edge of the continental landmass of northeast Asia, bordering North Korea in the north and surrounded by water on three sides. Its total area is 100,210 km^2^. Seventy percent of the country is mountainous, though the southern and western parts of the peninsula have more plains. Politically, it is divided into seven metropolitan and nine non-metropolitan provinces.

Korea features a temperate monsoon climate with cold winters, hot and humid summers, and sunny and generally dry springs and autumns. January is the coldest month, with a mean temperature ranging from −5 °C to 5 °C. Summer arrives in late May with warm, moist prevailing winds from the Pacific Ocean. Relative humidity is the highest in July at 80%–90% nationwide and the lowest in January and April at 30%–50%. Rainfall during the summer is characterized by heavy showers. The rainy season typically starts from late June and lasts until late July. 

### 2.2. Data Collection

We extracted the daily reported cases of AM from 1 January 2002 to 31 December 2012 from the national health insurance payment requests of the Health Insurance Review and Assessment Service database, which contains information on all insurance claims from about 97% of Korea population. Records contain information such as demographic characteristics, types of medical care institutions and treatment, medical expenses, healthcare service utilization, and disease classification codes based on the International Statistical Classification of Diseases and Related Health Problems 10th Revision while individual identifiers were completely anonymized. The daily meteorological and hydrological parameters, maximum, mean, and minimum temperatures (°C), relative humidity (%), amount of rainfall (mm), solar radiation (mJ/m^2^), and total hours of sunshine, were obtained from the Korea Meteorological Administration [28]. We averaged climate factors across all sites within each province for our analysis. The weekly averages of climate factors were calculated from the daily records. The sea surface temperature (SST) indexes used in this study were the Dipole Mode Index (DMI) and Nino3. The strength of the ENSO was measured using SSTs in the Nino3 region (5° S–5° N, 150°–90° W) of the Pacific Ocean. Nino3 values were taken from the National Oceanic and Atmospheric Administration climate prediction center [29]. The strength of the IOD is measured by the DMI, which is defined as the difference in SST between the western (10° S–10° N, 50°–70° E) and southeastern (10° S–0° S, 90°–110° E) tropical Indian Ocean. The dipole mode event is independent of the ENSO in the Pacific Ocean [16]. The DMI anomalies were derived from the Japan Agency for Marine-Earth Science and Technology [30]. Population data were retrieved from Statistics Korea [31]. The protocol of this study was approved by the institutional review board of Sungkyunkwan University (2015-05-010), and informed consent was wavered according to the Privacy Protection Act of the Republic of Korea.

### 2.3. Statistical Analysis

**Figure 1:**
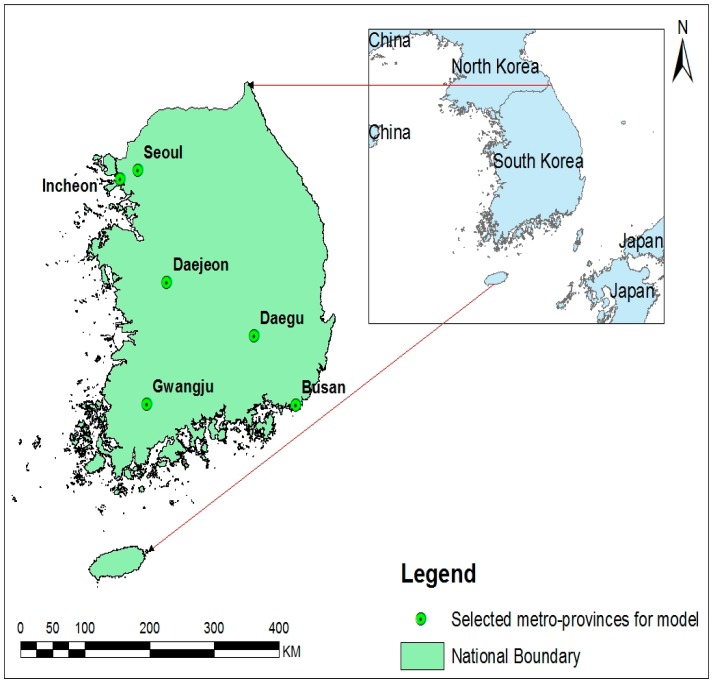
Map of geographical areas of Korea showing the six metropolitan provinces studied for model. The left panel shows the study sites in Korea map, and the right panel highlights the location of Korea in Asia.

We performed a descriptive analysis of the distribution of climate variables and AM cases. Annual incidences per 10,000 were estimated, and the number of weekly and monthly cases were plotted in each province against time series distributions of the average climate factors during the same period. We selected six metropolitan provinces for our study (Figure 1). In each metropolitan province, population was distributed in a relatively homogenous pattern with an absolute majority of Korean ethnicity. Climate variability was not great across the metropolitan provinces across the four distinct seasons. Several ecological studies have found associations between meteorological factors and enteroviral diseases with different lags [19,32,33]. Therefore, we designed the length of lag time in weeks after reviewing the incubation period of EVs [3] and other possible effects of host and environment on disease development. We used a maximum lag period of 13 weeks, with the current week set as lag 0. 

Univariate analysis was performed between the weekly averages of climate variables using Spearman correlation coefficients. In the analysis, we found a high correlation between humidity and rainfall as well as sunshine and solar radiation (*r_s_* ≥ 0.50). Thus, we did not include these pairs of climate variables in the same model to prevent multiple collinearity [33]. Among temperature measurements of mean, maximum, and minimum temperatures, the most statistically significant variable was selected for the analysis.

One sample Kolmogorov–Smirnov test was employed to verify that weekly AM cases did not fit a normal distribution. A generalized linear model (GLM) with a quasi-Poisson regression provided the best fit for the time series over-dispersed data of disease cases and meteorological factors [19]. Therefore, we fitted the multivariate quasi-Poisson regression models of weekly AM cases against meteorological factors. We adjusted each model separately to the year and the log annual province population as an offset. We calculated the quasi-Akaike information criterion (QAIC) values for each candidate model and compared the QAIC values of the models. The final model selected had the lowest QAIC values. We tested multi-collinearity among the explanatory variables using the variance inflation factor (VIF) [34] at an acceptable level (VIF ≤ 3, Tolerance ≥ 0.1). The model specifications we used for our GLM are as follows.

Log [E (Y)] = *β*_0_ + *β*_1_ (tm) + *β*_2_ (rn) + *β*_3_ (si) + *β*_4_ (log (Nino3)) + *β*_5_ (DMI) + *COV*s (1)

where E (Y) is the expected number of AM cases, *β*_0_ is the overall coefficient, and *β*_1_, *β*_2_, *β*_3_, *β*_4_ and *β*_5_ are coefficients for mean temperature (tm), rainfall (rn), solar radiation (si), Nino3 and DMI respectively. *COV*s are potential confounding factors, i.e., year and log annual population as an offset. Once a model was established, we applied it to the other five metropolitan provinces separately, estimating the final model by taking the averages from the respective weekly lags of individual variables in all six provinces.

We selected the specific lag of climate factors to examine the province-specific exposure–response relationship between the weekly count of AM cases and meteorological factors using a semiparametric generalized additive model (GAM) [35] with a log link function. To adjust the long-term trend and seasonal pattern in weekly morbidity, we chose a smoothing spline for time at 4 degrees of freedom (DF) per year. The model specification is given below.

Log [E (Y)] = *α* + *s* (local climate factors) + *DMI* + *s* (log (Nino3)) + *offset* (log (province population)) + *s* (*t*, DF = 4/year) (2)

where E (Y) is the expected number of AM cases, *α* is the intercept, *s* represents a smooth function using a smoothing spline, and local climate factors are mean temperature, rainfall, and solar radiation at lags of 0, 3, and 5 weeks, respectively. We used the DMI as a linear term and smoothing function of Nino3 at lag 11. 

We used the province-specific analyses for the following province-combined analyses. We pooled all province-specific data to construct a generalized additive mixed model (GAMM) and observed the variances between and within the provinces by applying a random effect as an indicator variable for any province in the model. The covariates and selected lags were the same in both the province-specific and province-combined models. To examine the major effect of climate factors on the development of AM, we also performed separate GAMM analyses for outbreak and non-outbreak years. All statistical analysis was performed using R 3.0.3, package ‘mgcv’ for GAM and package ‘gamm4’ for GAMM analyses [36].

## 3. Results

In 2002–2012, a total of 351,012 confirmed cases of AM occurred in Korea, with an average annual incidence of 6.6 per 10,000. The highest yearly incidence was 18.3 in 2002 followed by 12.7 in 2008, and the lowest incidence was 3.4 in 2007. Table 1 shows the general description of AM cases from 2002 to 2012 in Korea. 

The distributions of AM cases with meteorological variables show differences between the northern to southern provinces (Table 2). Generally, similar weather conditions were observed among the weekly average of climate factors in the six metropolitan political areas. The mean temperature was the highest in Daegu and the lowest in Incheon. Incheon also had the highest rainfall and the lowest solar radiation. The highest value of solar radiation was reported in Daejeon.

**Figure 2:**
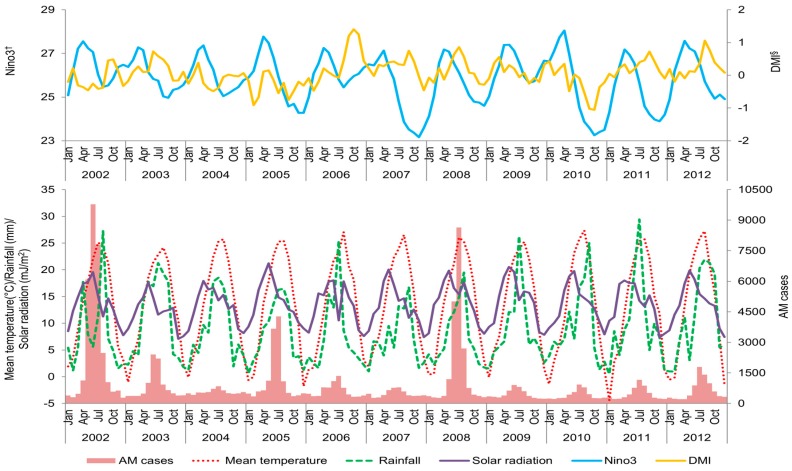
Monthly and yearly distributions of aseptic meningitis cases with meteorological factors in six metropolitan provinces of Korea, time period 2002–2012. The figure indicates monthly variation of aseptic meningitis cases with meteorological factors in the six metropolitan provinces of Korea over an 11-year period. The highest seasonal prevalence is found in the summer months. ^†^ monthly average of sea surface temperature in Nino3 region; ^§^ monthly average of Dipole Mode Index.

Figure 2 summarizes the monthly variations of AM cases with meteorological variables in six metro-provinces over the 11-year period. Although AM occurred throughout the year, the number of cases rose during the summer months and peaked in July. Nino3 peaked in April, and a marked variation was found in the DMI’s monthly peaks during the study period. 

**Figure 3:**
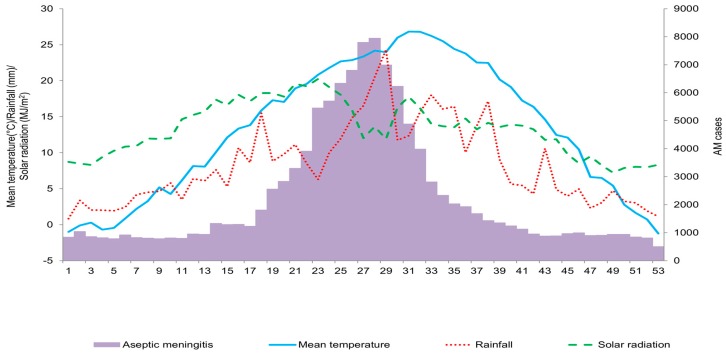
Weekly distribution of aseptic meningitis cases with local climate factors in six metropolitan provinces of Korea. The figure indicates weekly variation of AM cases with local climate factors among the six metropolitan provinces of Korea over the 11-year period. The highest weekly prevalence is found in the 28th week.

We also found weekly variations in AM with local climate factors among the six metropolitan provinces of Korea during the study period. Figure 3 reveals that AM patients were isolated every week, but the numbers fluctuated by week and peaked in the 28th week. Mean temperature peaked later while the solar radiation peaked earlier than the peak of AM. 

**Figure 4:**
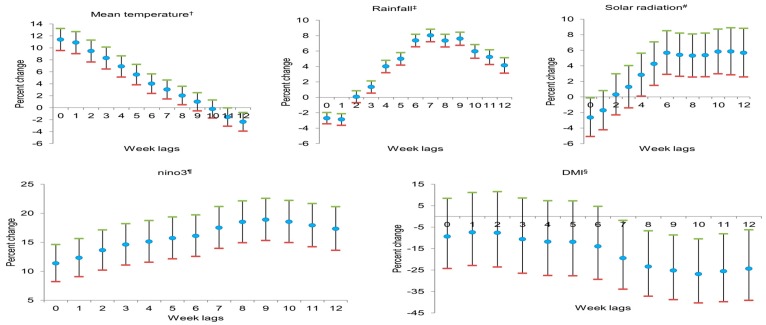
Quasi-Poisson regression model showing associations between climate variables and the weekly number of aseptic meningitis cases from 2002 to 2012 in six metropolitan provinces of Korea. ^†^ Weekly average of daily mean temperature (°C); ^‡^ weekly average of daily rainfall (mm); ^#^ weekly average of daily solar radiation (mJ/m^2^); ^¶^ weekly average of sea surface temperature in Nino3 region, ^§^ weekly average of Dipole Mode Index.

AM cases diagnosed at one week were associated with the weather variables that occurred during the same week and preceding weeks. Figure 4 shows the estimated single-week lag effects of mean temperature, rainfall, solar radiation, Nino3, and DMI based on a multivariate generalized linear quasi-Poisson regression adjusted for the total annual population and year. A 1 °C increase in mean temperature was associated with an 11.4% (95% confidence interval (CI) 9.6%–13.3%) maximum increase in AM cases in the same week (unlagged). A 10 mm increase in rainfall was associated with an 8% (95% CI: 7.2%–8.8%) maximum increase in AM cases after a 7-week lag. The estimated lagged effects of solar radiation were statistically significant for all lags except lags 1 to 3. A 1 mJ/m^2^ increase in solar radiation was associated with a 5.8% (95% CI: 3%–8.7%) increase in AM cases, maximized at a 10-week lag. Nino3 was positively associated with AM development in all lags. A 1 unit increase in Nino3 was associated with an 18.9% (95% CI: 15.3%–22.6%) maximum increase in AM cases at a 9-week lag. DMI showed significant negative associations from lag 7 and onwards. A 1 unit increase in DMI was associated with a maximum −26.8% (95% CI: −40.2%–−10.4%) decrease in AM cases at a 10-week lag. 

Percent change of risk and 95% CI were calculated by using a regression coefficient (*β*) and the following equation: percent change of risk = (exp[*β*] − 1) × 100 and 95% CI = (exp[*β*] − 1 ± 1.96 × standard error). Percent change of risk in mean temperature, solar radiation, and DMI indicates a change in AM cases for an increase of 1 unit; for rainfall, it indicates an increase of 10 unit. In Nino3, the percent change of risk for a 1 unit increase was ((1.01)^β^ − 1) × 100 and 95% CI = ((1.01)^β^ − 1 ± 1.96 × standard error).

**Figure 5:**
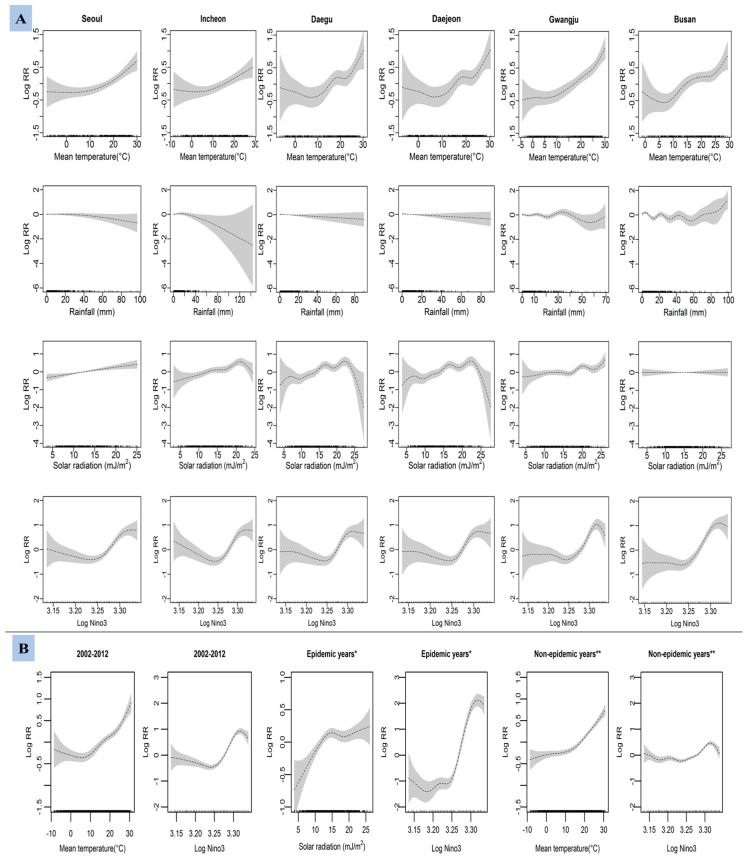
Generalized additive model for province-specific (**A**) and generalized additive mixed model for province-combined (**B**) associations in between the number of aseptic meningitis cases and weekly variations in climate factors in six metropolitan provinces of Korea from 2002 to 2012. * epidemic years (2002, 2005, and 2008); ** non-epidemic years (years excluding the outbreak years). Solids lines in each figure represents log relative risk (RR) at 95% confidence interval (shadows).

Figure 5 shows the province-specific and province-combined smoothed dose-response relationships between the weekly sum of AM cases and climate variables in various patterns, depending on locations and time intervals. In GAM models, the curves for mean temperature and Nino3 appear positive. We found variation in the associations of AM with rainfall and solar radiation. GAMM model reflects the overall positive associations of AM with mean temperature, solar radiation, and Nino3. During non-outbreak years, increasing mean temperature was positively associated with AM cases. Nino3 showed higher positive association with AM during outbreak years in comparison to non-epidemic years. 

## 4. Discussion

During the 11-year study period, AM cases in Korea varied every year. In particular, AM cases markedly increased in epidemic years [5,7,24,25,26], and each epidemic is characterized by the presence of certain predominant enteroviral serotypes [7,8,26,27]. The changing epidemiology and epidemics of AM could reflect improvements in public health, characteristics of the pre-existing etiologic agents, and changes in herd immunity from previous EV infections with protective antibodies [24]. 

In Korea, the number of AM cases and the associated incidence differ remarkably among the various provinces. In our study, AM cases varied seasonally in the 6 metropolitan provinces, occurring mostly in the summer months and peaking in July. This is similar to other Korean studies [7,24,25,26] and could be due to the factors such as viral strains, climate, season, geography, crowding, and socioeconomic status, which are shown to alter the prevalence and transmission of EVs in other countries [3,5,6,7]. Because the enteroviral diseases are most prevalent among children, it is also possible that outdoor play activities, which occur more often in summer than in winter, could facilitate EV transmission. 

Although several studies have found the effect of meteorological factors on the burden of hand-foot-and-mouth disease (HFMD) [32,33,37,38], and other infectious and non-infectious diseases [13,20,21], their effects on the development of AM remain obscure. We used a generalized linear quasi-Poisson model to estimate the lagged effects of diverse meteorological factors on AM cases in Korea using weekly data. These factors were included for the survival of pathogens in the external environment, seasonal fluctuations, human contact activities, and infection of humans. 

In our study, the estimated effect of a rise in weekly mean temperature on the number of AM cases was positive. Previous studies from temperate regions of China, Taiwan, and Japan also reported a similar effect of temperature on EV diseases [32,37,38]. An increase in the weekly lags of rainfall had a positive effect on AM cases. It is possible that rainfall could flush EVs into water bodies [6] that serve as a potential reservoir and vehicle, and thereby play an important role in infection. Solar radiation had a positive relationship with AM cases from lags 4 to 12. A recent study in Hong Kong also reported a positive effect of solar radiation on HFMD [39], which could be related to virus survival in the external environment that is influenced by variation in ultraviolet radiation, though the inactivation dose requirements vary by EV strains [11]. We found that IOD and ENSO had opposite effects on AM. An increase in the weekly lags of DMI was significantly associated to decrease to the AM after 6–12 weeks. In Korea, positive and negative IOD events form warm and dry, and cold and wet summers, respectively [40]. These climatic conditions might bring adverse effect for the enteroviral survival on the external environment. In Contrast, Cha (2007) found that DMI could not be associated with summer Korean climate variations such as ENSO [17]. Therefore, the effects of DMI on AM in Korea could be an area for future research. The significant positive associations between ENSO and AM cases in all lags suggest that ENSO is one of the main determinants for the weekly occurrence of AM in Korea. A relationship between oceanic SST indexes and other infectious diseases has been observed in several countries, including vector-borne dengue in Bangladesh and malaria in east African highlands [22,23], HFMD in Shenzhen, China [19], and other infectious diseases such as cholera in Bangladesh and influenza in Japan [20,21]. However, associations between SST indexes and AM have rarely been reported. 

We used the GAM and GAMM models to examine province-specific and province-combined dose-response relationships, respectively. The results suggest how local and oceanic climate variables affect AM distribution in 6 metro-provinces of Korea. Generally, the association between AM cases and meteorological variables reflects the biological plausibility. The prolonged survival of EVs depends on environmental characteristics, such as water sources, soil types, seasons, fomites, temperature, and relative humidity [1,10,33,37]. Weather conditions could be associated with changes in human contact and behavior. People are likely to spend more time out of the house in more crowded or air-conditioned environments when temperature and solar radiation are high during the summer months compared to when outdoor conditions are more moderate. This could lead to increases in contact frequency among persons that can accelerate EV transmission. We observed slight variation in the rainfall effect among the 6 provinces. In Singapore, weekly cumulative rainfall showed negative association with HFMD [41], while in Hong Kong, weekly average of rainfall showed positive association with HFMD [33]. Therefore, the effects rainfall on EV diseases depends on time intervals, locations, and amount, which could vary even among the regions of a single country. 

The largest periodic global climate variation is associated with two oceanic climate phenomena, i.e., ENSO and DMI [12,15,16], and they also affect Korean climate [17,18]. Several studies have documented a positive association between ENSO and HFMD in China [19], cholera epidemics in Bangladesh [20], and influenza epidemics in Japan [21]. We also found significantly higher positive associations between Nino3 and AM during outbreak years. In Korea, several studies have reported the effects of ENSO on variations in local temperature, rainfall, and precipitation [17,18], and these factors also influence the distribution of EV diseases in different countries [32,33,38]. 

In this study, the weekly count of AM cases in Korea tended to reach its highest level when the Nino3 value was the highest during epidemic years. It is possible that an increase in the weekly ENSO phenomenon affects the precipitation, temperature, and rainfall in the study area, which in turn influences the survival of EVs in the environment, thereby increasing the possibility of transmission [17,18]. Climate factors could also interact with each other in a given area [33]. The higher association of Nino3 with AM cases could result from interactions with other local climate factors in different periods. Therefore, due to complex interactions between weather indicators and ENSO, it is difficult to directly explain our observed association between Nino3 and AM occurrence during outbreak and non-outbreak years. Future studies on the effects of ENSO on population behavior, EV survival and susceptibility, and transmission of the disease pathogens could provide a better insight on such an association.

Our study investigated the effects of meteorological factors on weekly variation in AM cases in six metroplitan provinces of Korea using an ecological approach. This model can flexibly examine the possible weekly lag effects of weather conditions on variations in AM cases. Understanding the shapes of the dose-response relationships is important for environmental public health policy decision-making and setting weather quality standards. Comparison across the 6 metropolitan provinces is also important for demonstrating effects from one province to another.

Our study provides the first evidence of a relationship between weather variables, including SST indexes, and AM cases in Korea. A better understanding of those relationships, particularly the effect of Nino3 on EV ecology and host behavior, could provide an additional tool to predict epidemic risks.

This study has two major strengths. First, we applied a GLM to examine the lagged associations between the weekly number of AM cases and climate factors. Second, we investigated the effect of Nino3 and other climate factors on AM in 6 metropolitan provinces of Korea using GAMM and used a GAM to flexibly examine the concentration-response relationship in specific provinces. 

The present study also has limitations. This is an ecological study and cannot explore individual-level associations. Our analysis was mainly exploratory. Furthermore, we used data from only 6 metropolitan provinces of Korea, which are not representative of the whole country. 

## 5. Conclusions

We identified associations between AM cases and local and global climate factors in 6 metropolitan provinces of Korea. We found variations in the effect size by the location of the provinces and local climate variables. The effect of ENSO was more prominent during outbreak years than during non-outbreak years. This information can promote better preparedness and prevention measures in the study area before an actual upsurge in disease cases. 

## Figures and Tables

**Table 1:** General description of aseptic meningitis cases from 2002 to 2012 in Korea.

Provinces	Number	Incidence ^†^	Year	Number	Incidence ^†^
Seoul *	50,376	4.6	2002	87,212	18.3
Incheon *	11,395	3.9	2003	27,564	5.8
Daejeon *	9722	5.9	2004	17,981	3.7
Daegu *	11,904	4.3	2005	37,592	7.8
Gwangju *	15,045	9.4	2006	21,125	4.4
Ulsan	10,562	8.9	2007	16,508	3.4
Busan *	20,939	5.4	2008	62,111	12.7
Gangwon	14,190	8.7	2009	18,087	3.7
Gyeonggi	84,805	7	2010	18,686	3.8
Chungbuk	15,373	9.3	2011	20,557	4.1
Chungnam	11,411	5.2	2012	23,589	4.7
Gyeongbuk	8925	3.1			
Gyeongnam	54,686	15.8	Total	351,012	6.6
Jeonbuk	19,916	10			
Jeonnam	10,694	5.3			
Jeju	1069	1.8			

^†^: Cases per 10,000/year; * Selected metropolitan provinces for model.

**Table 2:** Weekly distribution of aseptic meningitis cases and average weather variables from 2002 to 2012 in six metropolitan provinces of Korea.

Province	Aseptic Meningitis Cases		Daily Mean Temperature (°C)		Rain Fall (mm/week)		Solar Radiation (mJ/m^2^)	
Provinces	x̄ ± SD	Range	x̄ ± SD	Range	x̄ ± SD	Max	x̄ ± SD	Range
Seoul	86.4 ± 139.9	5–1093	12.5 ± 10.2	−8.5, 30.2	9 ± 13.3	97.0	12.2 ± 4.4	2.9, 25.0
Incheon	19.6 ± 34.8	0–262	11.6 ± 9.8	−8.6, 28.8	8 ± 12.8	143.8	12.8 ± 4.7	3.2, 24.0
Daegu	20.4 ± 50.3	0–518	14.3 ± 9.4	−3.2, 30.9	7.0 ± 9.6	80.8	13.6 ± 4.5	4.0, 25.0
Daejeon	16.7 ± 34.4	0–318	12.8 ± 9.8	−6.8, 30.4	8.1 ± 10.8	89.9	13.7 ± 4.8	2.3, 28.0
Gwangju	25.8 ± 41.5	0–270	13.9 ± 9.3	−4.4, 30.2	8.3 ± 11.2	69.5	13.8 ± 4.7	4.0, 26.0
Busan	35.9 ± 69.2	0–641	14.6 ± 8	−1.7, 29.4	11.4 ± 15.2	99.5	14.2 ± 4.5	3.7, 27.0

x̄ ± SD: mean ± standard deviation.
